# Multi-center study on predicting breast cancer lymph node status from core needle biopsy specimens using multi-modal and multi-instance deep learning

**DOI:** 10.1038/s41523-023-00562-x

**Published:** 2023-07-13

**Authors:** Yan Ding, Fan Yang, Mengxue Han, Chunhui Li, Yanan Wang, Xin Xu, Min Zhao, Meng Zhao, Meng Yue, Huiyan Deng, Huichai Yang, Jianhua Yao, Yueping Liu

**Affiliations:** 1 Department of Pathology, The Fourth Hospital of Hebei Medical University, 050011 Shijiazhuang, Hebei China; 2 AI Lab, Tencent, 518057 Shenzhen, China; 3 Department of Pathology, Chengde Medical University Affiliated Hospital, 067000 Chengde, Hebei China; 4 Department of Pathology, Affiliated Hospital of Hebei University, 071000 Baoding, Hebei China; 5 Department of Pathology, Xingtai People’s Hospital, 054000 Xingtai, Hebei China; 6 Department of Pathology, First Hospital of Qinhuangdao, 066000 Qinhuangdao, Hebei China

**Keywords:** Breast cancer, Breast cancer

## Abstract

The objective of our study is to develop a deep learning model based on clinicopathological data and digital pathological image of core needle biopsy specimens for predicting breast cancer lymph node metastasis. We collected 3701 patients from the Fourth Hospital of Hebei Medical University and 190 patients from four medical centers in Hebei Province. Integrating clinicopathological data and image features build multi-modal and multi-instance (MMMI) deep learning model to obtain the final prediction. For predicting with or without lymph node metastasis, the AUC was 0.770, 0.709, 0.809 based on the clinicopathological features, WSI and MMMI, respectively. For predicting four classification of lymph node status (no metastasis, isolated tumor cells (ITCs), micrometastasis, and macrometastasis), the prediction based on clinicopathological features, WSI and MMMI were compared. The AUC for no metastasis was 0.770, 0.709, 0.809, respectively; ITCs were 0.619, 0.531, 0.634, respectively; micrometastasis were 0.636, 0.617, 0.691, respectively; and macrometastasis were 0.748, 0.691, 0.758, respectively. The MMMI model achieved the highest prediction accuracy. For prediction of different molecular types of breast cancer, MMMI demonstrated a better prediction accuracy for any type of lymph node status, especially in the molecular type of triple negative breast cancer (TNBC). In the external validation sets, MMMI also showed better prediction accuracy in the four classification, with AUC of 0.725, 0.757, 0.525, and 0.708, respectively. Finally, we developed a breast cancer lymph node metastasis prediction model based on a MMMI model. Through all cases tests, the results showed that the overall prediction ability was high.

## Introduction

Breast cancer is the most prevalent malignant cancer among women worldwide^1^. Observing the occurrence of axillary lymph node (ALN) metastasis in breast cancer patients is not only important for prognosis, but also for clinical diagnosis and treatment decisions^2,3^. Sentinel lymph node (SLN) is the first drainage site to experience the lymphatic spread of breast cancer. SLN biopsy (SLNB) is the standard method of ALN staging, which can guide clinicians in deciding axillary lymph node dissection (ALND), surgery, and follow-up treatment^4,5^. Preoperative prediction of lymph node status is critical for individualized treatment and for avoiding unnecessary surgery. Based on the idea of noninvasive prediction, several studies have attempted to utilize clinical predictors for establishing models to evaluate the possibility of SLN metastasis, and certain important prediction models have been developed. For instance, the most important prediction model is the Memorial Sloan–Kettering Cancer Center (MSKCC)^6^, which developed a nomogram to predict SLN metastasis. The ROC curve was 0.75, indicating an adequate level of prediction and discrimination. Liu et al. adopted the smote-bagged-tree algorithm to establish a model for predicting SLN metastasis in early breast cancer patients^7^. The ROC curve was 0.801, and the overall prediction ability was extremely high, indicating that the prediction model was accurate and stable.

Deep learning has achieved progress and application in the medical field^8–11^, yielding remarkable results in diagnosis and prognosis by automatically learning the latent features from medical data (i.e., histopathological images and clinical features)^12–16^. For example, Cao et al. developed a deep learning model to predict the microsatellite instability status, and interpreted the latent features by correlating with genomics and transcriptomics^17^. Liang et al. applied a deep learning model to clinical features at admission to predict the risk of COVID-19 patients developing critical illness^18^. Additionally, extensively utilizing various modal information has become an increasingly developed technology in the medical artificial intelligence (AI) field, and it has been widely demonstrated to be significantly useful^19–21^.

Multivariate logistic regression, tree-based methods, and shallow neural network-based methods have been used in previous studies to analyze clinical indicators^22–24^. Recent studies have shown that using tabular learning can effectively extract latent features and the interaction between previous methods on several tasks^25^. For histopathological image analysis, a weakly supervised method based on multi-instance learning can be used to learn latent features for subsequent analysis^26,27^.

Our research is based on the multi-modal prediction of clinicopathological indicators and pathological images, and makes full use of the clinicopathological information obtained from patients' core needle puncture samples before surgery. Therefore, we combined clinical pathological indicators with digital pathological images to establish a prediction model of breast cancer lymph node metastasis. This model performs a more accurate analysis for breast cancer, resulting in the improvement of the accuracy of clinical applications.

## Results

### Patients characteristics

In this study, the clinicopathological data and corresponding digital pathological images of 3701 female breast cancer patients were enrolled, with a mean age of 53 years. Patients were divided into training set (2222 cases), validation set (736 cases) and test set (743 cases). Among 3701 patients, according to postoperative pathological results and confirmed by immunohistochemical results, 1953 patients had no lymph node metastasis, 118 patients were isolated tumor cells (ITCs), 564 were micrometastasis, and 1066 were macrometastasis. There was no significant difference in clinicopathological features among the three cohorts (*P* > 0.05) (Table 1).

**Table 1:** Patient and tumor characteristics of training set, validation set and test set.

Characteristic	Total	Train	Val	Test	*P* value
No.	3701	2222	736	743	
Menopause status					0.998
premenopausal	1522 (41%)	897 (40%)	311 (42%)	314 (42%)	
menopause	2179 (59%)	1325 (60%)	425 (58%)	429 (58%)	
Tumor size					0.074
≤2 cm	1921 (52%)	1147 (52%)	368 (50%)	406 (55%)	
å 2 cm	1780 (48%)	1075 (48%)	368 (50%)	337 (45%)	
Histological grade					0.409
1	227 (6%)	153 (7%)	34 (5%)	40 (5%)	
2	2616 (71%)	1562 (70%)	536 (73%)	518 (70%)	
3	858 (23%)	507 (23%)	166 (23%)	185 (25%)	
Lymph node status					0.052
negative	1953 (53%)	1173 (53%)	387 (53%)	393 (53%)	
ITCs	118 (3%)	67 (3%)	29 (4%)	22 (3%)	
micrometastasis	564 (15%)	340 (15%)	95 (13%)	129 (17%)	
macrometastasis	1066 (29%)	642 (29%)	225 (31%)	199 (27%)	
Tumor location					0.558
UOQ	2090 (56%)	1251 (56%)	427 (58%)	412 (55%)	
UIQ	865 (23%)	511 (23%)	176 (24%)	178 (24%)	
LOQ	441 (12%)	276 (12%)	74 (10%)	91 (12%)	
LIQ	305 (8%)	184 (8%)	59 (8%)	62 (8%)	
Vasular invasion					0.131
absent	2975 (80%)	1774 (80%)	609 (83%)	592 (80%)	
present	726 (20%)	448 (20%)	127 (17%)	151 (20%)	
Nerve invasion					0.824
absent	3199 (86%)	1936 (87%)	627 (85%)	636 (86%)	
present	502 (14%)	286 (13%)	109 (15%)	107 (14%)	
ER					0.874
negative	781 (21%)	466 (21%)	158 (21%)	157 (21%)	
positive	2920 (79%)	1756 (79%)	578 (79%)	586 (79%)	
PR					0.600
negative	957 (26%)	584 (26%)	190 (26%)	183 (25%)	
positive	2744 (74%)	1638 (74%)	546 (74%)	560 (75%)	
HER2					0.965
negative	2625 (71%)	1577 (71%)	522 (71%)	526 (71%)	
positive	1076 (29%)	645 (29%)	214 (29%)	217 (29%)	
Ki67					0.234
<20%	531 (14%)	320 (14%)	113 (15%)	98 (13%)	
≥20%	3170 (86%)	1902 (86%)	623 (85%)	645 (87%)	
TILs					0.568
low	2657 (72%)	1572 (71%)	543 (74%)	542 (73%)	
middle	768 (21%)	481 (22%)	145 (20%)	142 (19%)	
high	276 (7%)	169 (8%)	48 (7%)	59 (8%)	
BI-RADS grade					0.458
4a	1032 (28%)	615 (28%)	193 (26%)	224 (30%)	
4b	679 (18%)	431 (19%)	119 (16%)	129 (17%)	
4c	1052 (28%)	617 (28%)	224 (30%)	211 (28%)	
5	938 (25%)	559 (25%)	200 (27%)	179 (24%)	
Molecular subtype					0.451
Luminal	2931(79%)	1762(79%)	580(79%)	589(79%)	
HER2 over-expression	580(16%)	344(15%)	115(16%)	121(16%)	
TNBC	190(5%)	116(6%)	41(5%)	31(5%)	

*P* values < 0.05 were considered statistically significant, and all *P* values were two-sided. *P* value is obtained by comparing the validation set and testing set.

*UOQ* upper outer quadrant, *UIQ* upper inner quadrant, *LOQ* lower outer quadrant, *LIQ* lower inner quadrant.

### The characteristics of the deep learning model of clinicopathological parameters, WSI and MMMI

**Fig. 1:**
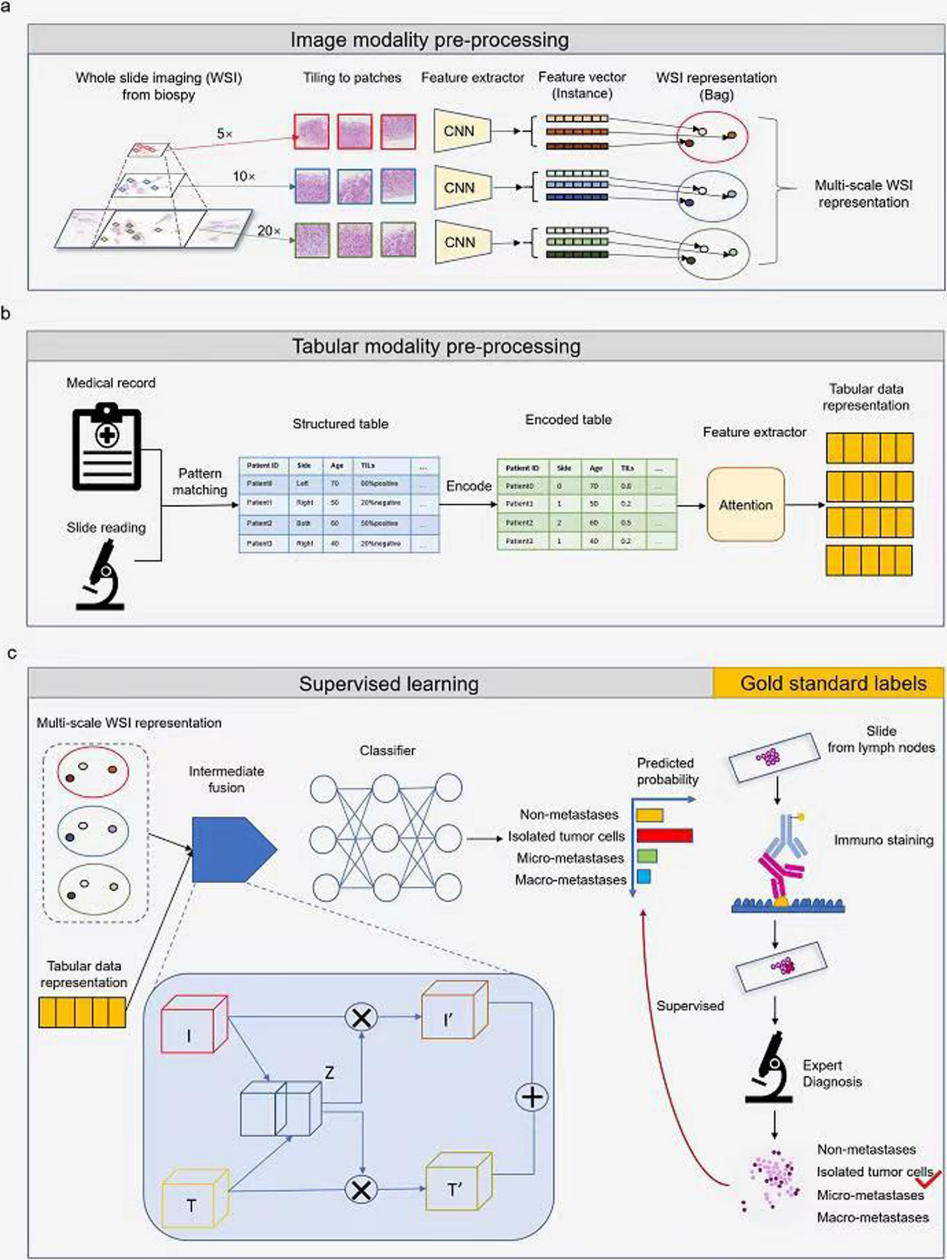
Model development overview. The model development can be categorized into three parts, the preprocessing of image modality data, the preprocessing of tabular data, and training of the classification model. **a** Image modality preprocessing. The whole slide imaging from the biopsy was annotated with cancer regions and then tiled into patches at different scales (5×, 10×, and 20×). The EfficientNet pre-trained on ImageNet dataset36 was applied to extract features from each patch. All the patches inside one WSI can be combined as WSI-level representation. Since the prediction on WSI using patch-level features can be formulated as a multi-instance learning problem, the patch-level feature vectors can be considered as instances, while WSI-level representation is a bag containing all the instances. Each scale can be processed in the same pipeline. **b** Tabular modality pre-processing. Medical records and clinicoparameters were obtained from the hospital system and slide reading by experts. Both of them are in semi-structured natural language descriptions. A set of matching rules based on regular expression were applied to extract the structured information. Then we encoded the category variables and inputted the encoded table into a feature extractor to generate tabular data representation. **c** Supervised learning and gold standard labels. Then the fused cross-modality representation was processed by a classification network to produce the probability of non-metastases, isolated tumor cells, micro-metastases, and macro-metastasis. This part was trained end-to-end and was supervised by the gold standard labels generated by expert diagnosis on IHC stained slide of lymph node after surgery.

For histopathological images, we used multi-instance learning (MIL) method to analyze the characteristics of the whole slice images (WSIs). The cancer tissues are first extracted from each WSI and are tiled into patches for further feature extraction and MIL analysis. As for the clinicopathological parameters, we applied tabular learning model to learn the interaction between the features and abstract the final representation of the tabular data by nonlinear combination of the features. The clinicopathological parameters are first extracted from the tabular recording and transformed into structured data. The pre-processing of both modalities is conducted automatically and effectively for model analysis. After effective pre-processing, data cleaning and imputation, we developed a novel modal fusion module that aims at borrowing information from clinicopathological parameters to focus on discriminative patches in multi-instance learning of the histopathological images, and promoting the flow of complementary information between modalities through intermediate fusion (Fig. 1). The novelty of the proposed modal fusion module lies in three folds: 1) using the information of clinicopathological parameters to guide the multi-instance learning process of histopathological images; 2) employing multi-scale histopathological images for comprehensive analysis from different scales; 3) generating cross-modal represent by capturing the relation of different modalities and recalibrating the informative features in each modality.

### Predictive performance of lymph node metastasis model

**Fig. 2:**
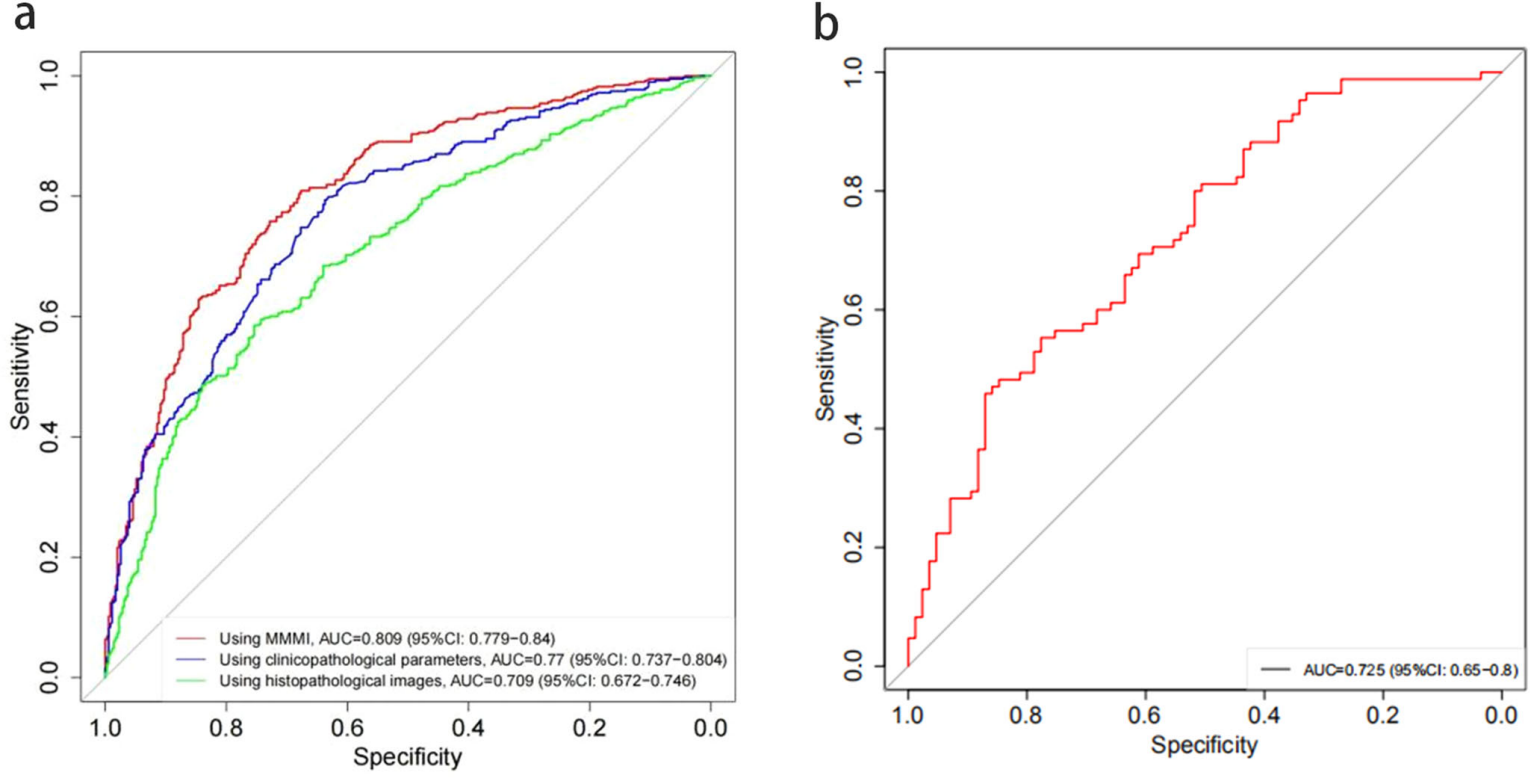
Prediction performance of different models for predicting breast cancer lymph node status(no metastasis, metastasis). **a** The test set showed that MMMI can predict lymph node metastasis more accurately than based on single clinicopathological factors or WSI features. **b** The external validation set confirmed the performance of MMMI model in predicting lymph node status.

The MIL model was evaluated for prediction of lymph node status (no metastasis and metastasis) in the test set. The area under curve (AUC) of MIL model for clinicopathological features was 0.770, WSI was 0.709, and MMMI was 0.809. MMMI developed by combining clinicopathological features with WSI showed a more accurate prediction accuracy for lymph node status prediction (Fig. 2).

**Fig. 3:**
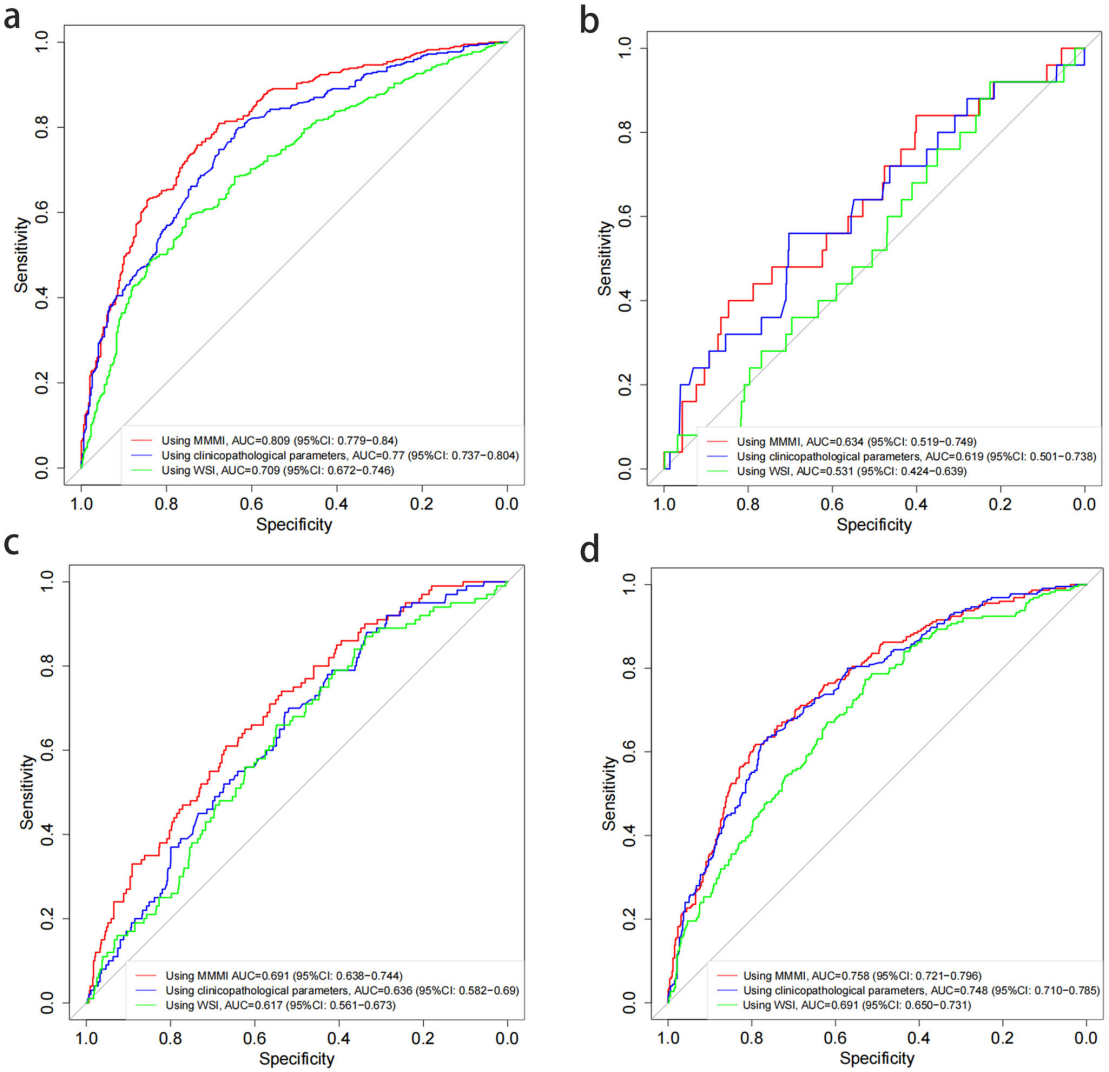
Prediction performance of different models for predicting breast cancer lymph node status(no metastasis, ITCs, micrometastasis and macrometastasis). The AUC values of lymph node status were predicted by MMMI, clinicopathological parameters deep learning model and WSI deep learning model. The red line means MMMI. The blue line means clinicopathological parameters. The green line means WSI. **a** No metastasis. **b** ITCs. **c** Micrometastasis, **d** Macrometastasis. No matter which kind of lymph node state is predicted, the prediction result of MMMI based on the combination of clinicopathological parameters and WSI is obviously higher than that of single model based on clinicopathological indicators or WSI.

In order to predict lymph node status more accurately and provide a more detailed basis for clinical decision, we classified lymph node status in more detail (no metastasis, ITCs, micrometastasis and macrometastasis). For metastasis-free, predicted by deep learning model of tabular, the AUC was 0.770 (95%CI: 0.737–0.804), accuracy was 0.723, sensitivity was 0.791, and specificity was 0.649. Predicted by deep learning of WSI, the AUC was 0.709 (95%CI: 0.672–0.746), accuracy was 0.669, sensitivity was 0.593, and specificity was 0.757. Predicted by MMMI, the AUC was 0.809 (95%CI: 0.779–0.840), accuracy was 0.751, sensitivity was 0.768, and specificity was 0.734. In contrast, MMMI demonstrated better prediction performance. The same results were found in ITCs, micrometastasis and macrometastasis (Table 2). Finally, no matter which kind of lymph node status was predicted, the prediction of MMMI was obviously better than that of single model based on clinicopathological features or digital pathological images. The ROC curves were shown in Fig. 3A–D.

**Table 2:** Performance comparison of different models for predicting lymph node status.

Class	Methods	AUC	ACC	SEN	SPE
Negative	Tabular	0.770(0.737–0.804)	0.723(0.693–0.752)	0.791(0.662–0.845)	0.649(0.580–0.769)
	MIL-WSI	0.709(0.672–0.746)	0.669(0.637–0.703)	0.593(0.458–0.728)	0.757(0.617–0.874)
	MMMI	0.809(0.779–0.840)	0.751(0.720–0.779)	0.768(0.616–0.855)	0.734(0.637–0.874)
ITCs	Tabular	0.619(0.501–0.738)	0.701(0.265–0.938)	0.600(0.240–0.960)	0.705(0.241–0.962)
	MIL-WSI	0.531(0.424–0.639)	0.346(0.230–0.938)	0.880(0.200–1.000)	0.329(0.205–0.964)
	MMMI	0.634(0.519–0.749)	0.746(0.392–0.880)	0.600(0.320–0.960)	0.751(0.375–0.897)
Micrometastasis	Tabular	0.636(0.582–0.690)	0.538(0.351–0.743)	0.770(0.450–0.960)	0.508(0.261–0.787)
	MIL-WSI	0.617(0.561–0.673)	0.490(0.380–0.682)	0.800(0.510–0.930)	0.440(0.302–0.706)
	MMMI	0.691(0.638–0.744)	0.623(0.431–0.773)	0.710(0.450–0.910)	0.611(0.355–0.818)
Macrometastasis	Tabular	0.748(0.710–0.785)	0.723(0.638–0.759)	0.658(0.582–0.827)	0.757(0.568–0.807)
	MIL-WSI	0.691(0.650–0.731)	0.616(0.552–0.692)	0.769(0.542–0.871)	0.544(0.415–0.747)
	MMMI	0.758(0.721–0.796)	0.734(0.647–0.773)	0.653(0.556–0.822)	0.776(0.591–0.844)

### Predictive performance of lymph node status in different molecular subtypes

**Fig. 4:**
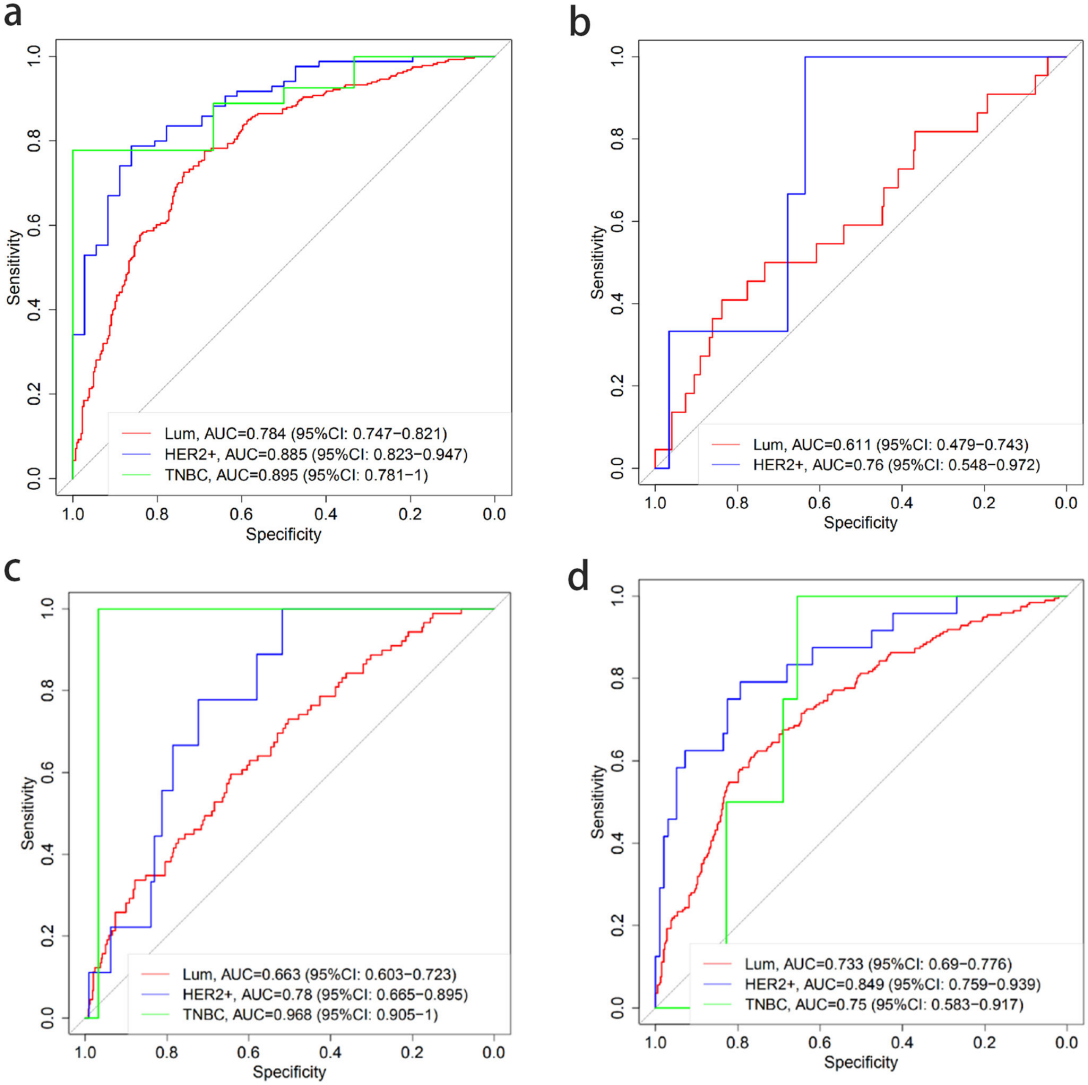
Prediction performance of MMMI model with breast cancer different molecular subtypes. The AUC value of lymph node status of each molecular subtype was predicted by using MMMI. The red line means Luminal subtype. The blue line means HER2 over-expression subtype. The green line means TNBC subtype. **a** No metastasis. Compared with the other two subtypes, TNBC has the best predictive performance, and the AUC of 0.895. **b** ITCs. HER2 over-expression subtype has a good predictive performance, and the AUC of 0.76. **c** Micrometastasis. TNBC has the best predictive performance, and the AUC of 0.968. **d** Macrometastasis. HER2 over-expression subtype has the best predictive performance, and the AUC of 0.849.

In addition, we also analyzed the performance of the MMMI model in predicting lymph node metastasis of different molecular subtypes. The AUC of different lymph node status in Luminal breast cancer were 0.784 (95%CI: 0.747–0.821), 0.611 (95%CI: 0.479–0.743), 0.663 (95%CI: 0.603–0.723) and 0.733 (95%CI: 0.69–0.776), for no metastasis, ITCs, micrometastasis, and macrometastasis, respectively. The AUC in HER2 over-expressed group were 0.885 (95% CI: 0.823–0.947), 0.76 (95% CI: 0.548–0.972), 0.78 (95% CI: 0.665–0.895), 0.849 (95% CI: 0.65–0.895), for no metastasis, ITCs, micrometastasis, and macrometastasis, respectively. The AUC in TNBC were 0.895 (95% CI: 0.781–1), 0.968(95% CI: 0.905–1) and 0.75 (95% CI: 0.583–0.917), respectively. Due to the limitation of ITCs samples, the AUC results of ITCs were not obtained in the TNBC group. However, by comparing all results, we found that MMMI demonstrated a better prediction accuracy no matter which kind of lymph node status, especially in the molecular subtype of TNBC, and the ROC curve was shown in Fig. 4.

### Feature importance analysis

**Fig. 5:**
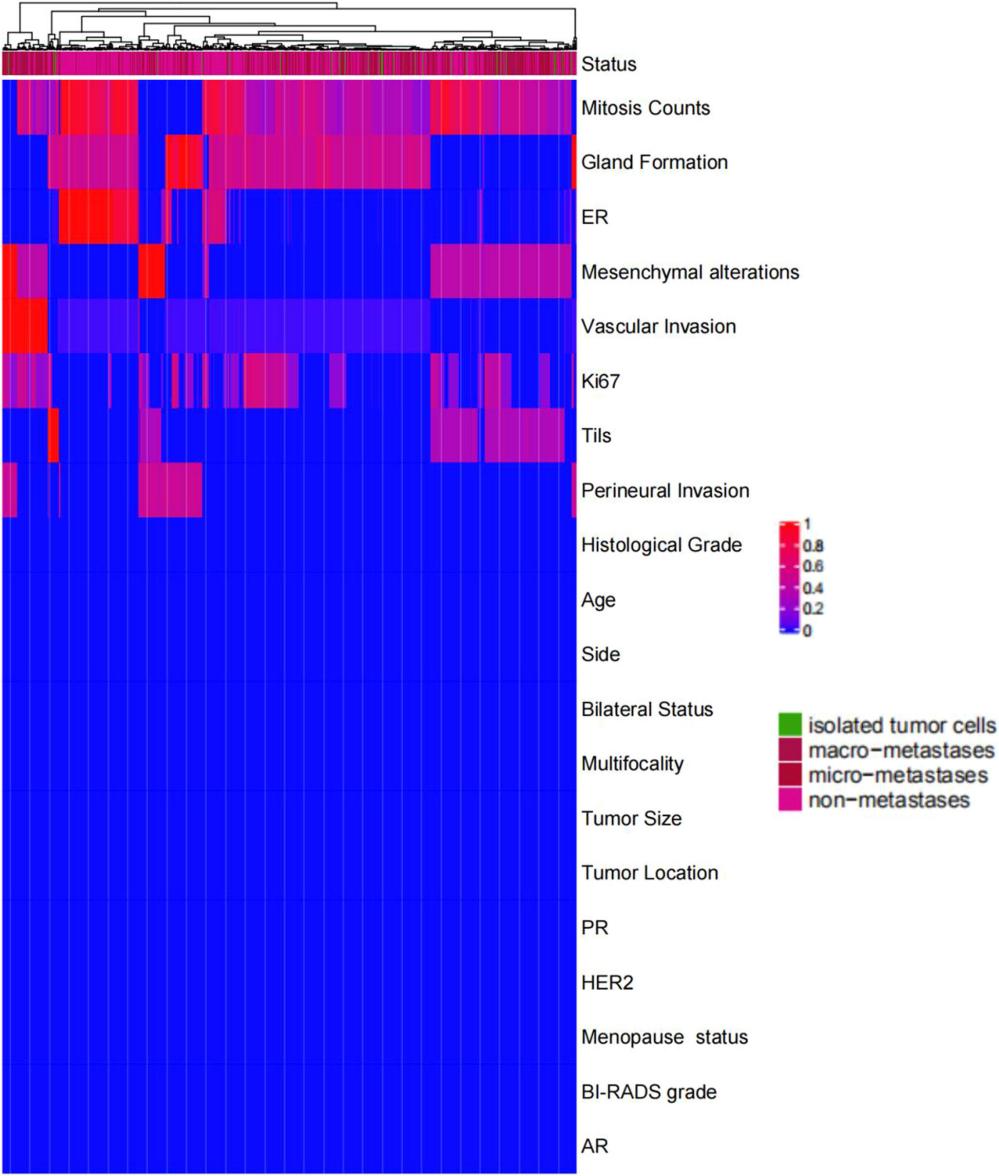
Feature importance analysis. Through comprehensive analysis of clinicopathological features, the weight of each factor in the prediction of lymph node metastasis was calculated. The results showed that pathological mitosis had the highest weight in breast cancer lymph node metastasis, and the other factors with higher weight were gland formation, ER, stoma changes, vascular invasion, Ki67, and TILs.

We explored the feature importance based on how informative each feature is for prediction. The analysis results showed that the characteristics of mitosis, glandular ducts and vascular invasion played an important role in predicting lymph node metastasis (Fig. 5).

### Testing of external data sets (multi-center study)

**Fig. 6:**
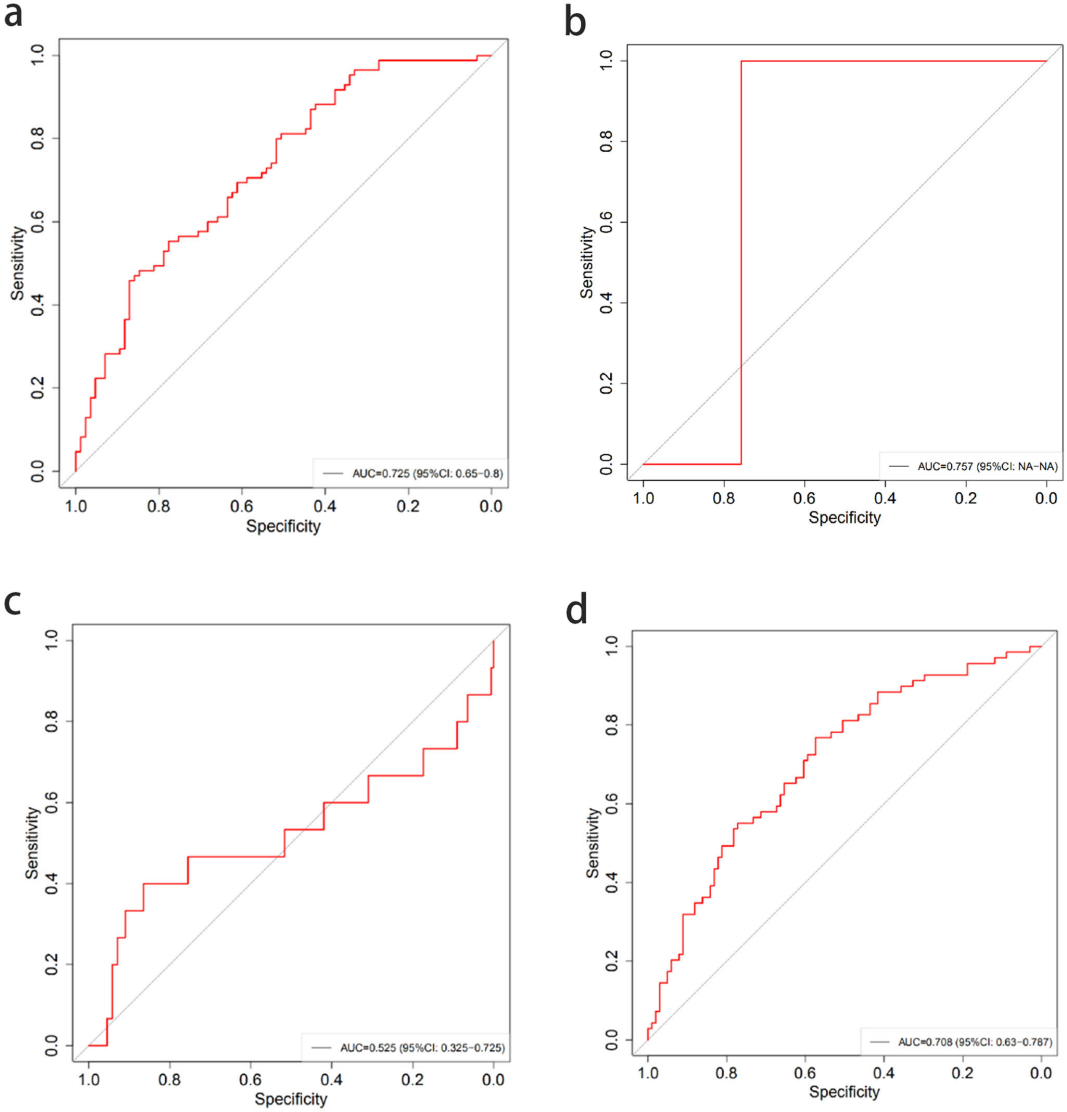
Prediction performance of MMMI model for external validation. MMMI was used to predict lymph node metastasis using external data. **a** No metastasis. **b** ITCs. **c** micrometastasis. **d:** Macrometastasis. In the external validation set, MMMI also achieved good results in predicting lymph node status, with AUC of 0.725, 0.757,0.525 and 0.708, respectively. Except for the low AUC value of micrometastasis due to the number of cases, the other groups showed higher prediction performance.

In order to test the applicability of MMMI, we collected 190 external validation cohorts from four medical centers in Hebei Province for external validation, including 107 without lymph node metastasis, 15 with ITCs, 8 with micrometastases and 58 with macrometastases. In the external validation sets, MMMI also showed better prediction accuracy in the four classification, with AUC of 0.725 (95% CI: 0.65–0.8), 0.757 (95% CI: Na–Na), 0.525 (95% CI: 0.325–0.725), 0.708 (95% CI: 0.63–0.787), respectively. (Fig. 6). Except for the low AUC value of micrometastasis due to the number of cases, the others showed higher prediction performance.

## Discussion

ALN metastasis of breast cancer not only determines the method of operation, but is also an important prognostic factor. Accurate prediction of lymph node metastasis in breast cancer patients can assist clinicians to develop axillary lymph node dissection, reduce postoperative complications, and improve prognosis. Previous studies^28–30^ predicted ALN status from clinicopathological data, such as tumor grade, tumor size, lymphatic vascular invasion, etc. However, these studies only predicted the presence or absence of lymph node metastasis and could not distinguish between ITCs, micrometastases, or macrometastases. Moreover, the histopathological image provides the tumor micro-environment that can not be quantified or fully described in the medical report. We can only make a semi-quantitative evaluation through microscopic observation. In this regard, we propose to utilize the tabular modality information to guide the patch feature aggregation procedure. Through explicitly leveraging the multi-modality fusion information in a cross-modal manner, the global feature can be complemented for better instance-wise attention.

Deep learning has gained increasing attention in the field of medical imaging. Currently, deep convolutional neural networks (DCNNs) are one of the well-known types of deep learning algorithms. DCNNs are widely used in medical image processing and pattern recognition because of their simple structure and strong applicability, especially in imaging and pathology^31^. In a previous study, researchers successfully developed a prediction model for lymph node metastasis in breast cancer patients using a deep learning neural network. The AUC of the CNN model with the best performance was 0.89. Additionally, the ROC performance of this model was better than that of the three experienced radiologists. These results demonstrated the feasibility of using CNNs to predict whether early primary breast cancer will metastasize and determine the feasibility of using deep learning methods to predict clinically negative ALN metastasis from ultrasound images in patients with primary breast cancer^32^. A deep learning radiography (DLR) method based on clinical parameters of breast conventional ultrasound (CUS) and shear wave elastography (SWE) images has been developed and verified^33^, which can be used to predict the ALN status of clinical T1 or T2 breast cancer patients before surgery. The differential diagnosis effect of this method on axillary negative (N0) and axillary metastasis (*N* + (≥1)) is better than that of the single method. Furthermore, the model indicated high discrimination between patients with low risk of axillary metastasis (*N* + (1–2)) and high risk (*N* + (≥3)).

In clinical practice, an increasing number of clinicians wish to understand the SLNs state before undergoing surgery, so as to guide the operation method. The prediction results obtained using these models are more reliable than simple clinical estimates. In our study, four classifications of lymph node metastasis can be accurately predicted using preoperative multi-modal data, combined with clinicopathological indexes and pathological image features. For patients with different metastases, providing targeted surgical methods can avoid over-treatment and improve the quality of patients’ lives. However, some information in HE stained slices, such as the tumor micro-environment, cannot be quantified. Deep learning can be used to extract more information about the tumor micro-environment, such as tumor-infiltrating lymphocytes (TILs) from pathological images. These two methods reflect the patients’ information at different levels, and when combined, they provide a more comprehensive representation of the patient’s condition and disease progression. There are several studies on lymph node metastasis in imaging that have obtained some effect currently^34–36^. However, the detection of imaging focuses on macroscopic features, and it is easy to miss the detection of early small metastases such as micrometastases or ITCs. In this study, we propose a novel MMMI joint learning model with a Multi-modal Multi-instance Fusion (M3IF) module that can generate a cross-modal representation of different modalities to recalibrate the features in each modality and capture the relation between different modalities, alleviating the impact of the data misalignment between modalities. We leverage the cross-modal representation to guide the attention-based MIL to strengthen the attention on informative instances in image modality. Multi-scale images provide a more comprehensive representation of the image modality. We predicted the status of lymph nodes (no metastasis, ITC, micrometastasis and macrometastasis) and compared the prediction efficiency of the models. The results showed that the MMMI model had better prediction ability than the single model. Evaluation of the model on different molecular subtypes of breast cancer were performed, and the results showed that MMMI could predict the lymph node status of each group, especially TNBC. The deficiency of this study was that the number of ITCs and micrometastases cases was small, but MMMI still showed good predictive ability. We will further expand the amount of data for increasing convince.

To test the applicability of the MMMI, we selected 190 cases for multi-center verification. For predicting the presence of lymph node metastasis, the AUC value was 0.6258. In addition, we tested the performance of the model for predicting no metastasis, ITCs, micrometastases, and macrometastases. Except for the low AUC value of micrometastases due to the number of cases, the other groups showed highly predictive performance. The performance of the model declined in external validation because of the differences in interpretation between different centers and the influence of HE staining. The model can be improved by adding external data, unifying interpretation and marking, and optimizing the WSI.

This study has some limitations. There was no predictive verification of ITCs in TNBC due to the few cases of ITCs and uneven distribution, and an excessive AUC value was observed in other molecular classifications. Although MMMI can predict lymph node metastasis more accurately than single clinicopathological factors or WSI features, it has a certain decline in the four classifications of lymph node metastasis. In the future, we plan to optimize MMMI by increasing the sample size, adding other central sample data or gene test results to obtain more accurate and detailed prediction results of lymph node status.

## Methods

### Patients

We collected the clinicopathological data and pathological images of preoperative core needle biopsy of 4038 female invasive breast cancer patients in the Fourth Hospital of Hebei Medical University from January 2015 to December 2018. Additionally, the clinicopathological data and whole slide imaging (WSIs) of 190 female invasive breast cancer patients from four medical centers in Hebei Province were collected for external validation of the proposed method. The study protocol was reviewed and approved by the ethics committee of The Fourth Hospital of Hebei Medical University (approval no. 2022KY059). The study was performed in accordance with the ethics standards of the participating institutions and the tenets of the Declaration of Helsinki. All participants provided written informed consent to take part in the study. All hematoxylin-eosin (HE) staining slices were scanned by PRECICE 600 fully Automatic Digital Slice Scanner (Chongqing, China). The inclusion criteria were as follows: (1) three experienced pathologists confirmed that all breast biopsy specimens were invasive breast cancer; (2) no neoadjuvant treatment (NAT) pre-operation was performed; (3) histopathology and immunohistochemistry were used to postoperatively confirm lymph node metastasis; and (4) complete clinical pathological data was obtained. The exclusion criteria were as follows: (1) microinvasive carcinoma (invasive lesions < 1 mm); (2) special types of invasive carcinoma; (3) poor/blurred scanned pathological image quality; (4) preoperative treatment (NAT, chemotherapy, radiotherapy and chemotherapy, ablation, etc.); and (5) incomplete clinical pathological data. Finally, 3701 patients were selected for this study.

Patients’ clinicopathological data of biopsy tissues were collected and evaluated, including age, menopausal status, tumor size, histological grade, nuclear atypia, mitosis counts, TILs, histological grade, ER (estrogen receptor) status, PR (progesterone receptor) status, HER2 (human epidermal growth factor receptor 2) status, lymph node metastasis postoperatively.

### Pathological evaluation

In AJCC, lymph node metastasis can be divided into ITCs (≤200 scattered tumor cells or tumor clusters ≤ 0.2 mm), micrometastasis (tumor > 0.2 mm and ≤2 mm), and macrometastasis (tumor > 2 mm)^37^, according to the number of cancer cells in metastatic lymph nodes and the size of the tumor focus. Histological grading was based on the World Health Organization classification of breast tumors (5th Edition)^38^ and the Nottingham grading system. All cases were classified as grade I, grade II, or grade III. TILs evaluation criteria: area occupied by mononuclear inflammatory cells over total stromal area^39,40^. More than 1% of positive tumor cell nuclei are considered hormone receptor-positive for ER and PR. IHC (Immunohistochemistry) score of 3+ or FISH (Fluorescence in situ hybridization) amplification was defined HER2 positivity. All cases divided into three subtypes: luminal (hormone receptor-positive, including luminal A and luminal B), HER2 over-expression (hormone receptor negative, HER2 positive), and triple negative breast carcinoma (both hormone receptor and HER2 negative, TNBC).

Immunohistochemical studies were performed on 4-µm formalin-fifixed paraffifin embedded (FFPE) tissues sections with commercially available antibodies targeting the following proteins: ER (working fluid, clone Sp1; Roche), PR (working fluid, clone 1E2; Roche), HER2 (working fluid, clone 4B5; Roche), Ki67 (working fluid, clone 30-9; Roche). HER2 fluorescence in situ hybridization was performed by using Path Vysion HER-2 DNA Probe Kit (Abbott, USA).

### Structure and standardization of the data

Clinicopathological parameters were extracted from this report using a text pattern-matching algorithm. For the categorical variables, the LabelEncoder function in the scikit-learn package was used to encode the target categorical variables into numerical variables. Thus, our algorithm generated structured data for each patient. Multivariate imputation via chained equations was applied to impute missing data^41^. Color normalization was performed on all scales of histopathological images using an enhanced cycle-consistent generative adversarial network^42^.

### Data partitioning, image preprocessing, and data augmentation

The dataset was stratified at the patient level and randomly divided into training (60%), validation (20%), and test (20%) sets. Given the large size (typically 130,000 × 50,000 pixels) of a WSI, the WSIs were tiled into 512 × 512 patches in the form of a grid for subsequent processing. In this study, three magnification scales (5×, 10×, and 20×) were explored, under which tiling was performed^43^. The threshold of overlap varied among different magnifications. That is to say, three sets of patches (size 512 × 512) corresponding to each scale (5× 10×, and 20×) are tiled separately under each scale. The features of each set of patches are extracted separately to get local and global pathological information for further cross-modal fusion. Data augmentation (including rotating, flipping, changing brightness and contrast) was applied to the patches during the training process to improve the generalization.

### Development, validation and interpretation of the model

Deep learning, as a form of representation learning, transforms raw data into a suitable representation for pattern recognition in specific tasks^44^. In this study, we first generate representation of WSI and clinicopathological parameters with specific deep learning model, respectively. And then we generate the cross-modal fusion features with innovatively designed multimodal integration module. The cross-modal fusion futures are then employed to recalibrate the representation for more efficient representation of each modal. Finally, we generate the final multi-modal output based on the recalibrated features from each modality. Then the established model is trained, validated, and interpretated. The entire process is elaborated through the following steps: (1) Generation of MIL representation of WSI; (2) Generation of tabular learning-based representation of the clinicopathological parameters; (3) Generation of cross-modal fusion features by integrating the representation of WSI and clinicopathological parameters; (4) Recalibration of MIL representation based on cross-modal fusion features; (5) Recalibration of tabular learning-based representation of the clinicopathological parameters based on cross-modal fusion features; (6) Fusion of the recalibrated features for final prediction; (7) Model training and validation; 8) Model interpretation. The codes of the model building are publicly available and the open source link could be found in the CODE AVAILABILITY section.

#### Generation of MIL-based representation of WSI

Each WSI was tiled into patches, and the prediction of lymph node metastasis (LNM) relies on the entire Region of Interest (ROI) of WSIs instead of individual patches^45^. EfficientNet^46^ pre-trained on the ImageNet dataset^47^ was applied to extract patch-level features, and attention layers on the instance level and feature level were applied as the WSI modality network backbone. In this way, the embedding of patch-level feature was regarded as the representation of the WSI. The contribution of instances and features would be reweighed during training process, respectively.

#### Generation of tabular learning-based representation of the clinicopathological parameters

We adopted an the TabNet encoder from attentive interpretable tabular learning network, TabNet^25^, to generate a representation of the clinicopathological parameters. The encoder is composed of a feature transformer, an attentive transformer and feature masking. The network employed sequential attention on features for inference in each decision step and learned the salient features from the structured clinicopathological parameters.

#### Generation of cross-modal fusion features by integrating the representation of WSI and clinicopathological parameters

We developed a new multi-modal multi-instance (MMMI) fusion module comprising multi-modal joint instance aggregate learning and global-aware instance aggregation. The representation of WSIs and clinicopathological parameters were input to the module and embedded as the global multi-modal feature, which was used to guide the learning process of each modality in turn.

To be specific, in the pathological image part, the image features are extracted from the image under three magnifications. Because the ROI size under different magnifications is inconsistent, and the ROI size of different patients is different, the number of features extracted in this step will vary greatly. Therefore, an effective feature selection mechanism is needed to retain effective sample features. At the same time, this method uses data from multiple modalities, and it also needs to effectively integrate information from different modalities to jointly complete predictions. The multi-modal multi-instance module will solve the aforementioned problem of feature selection and modal information integration, there will be an indefinite set of image features at each magnification. By means of global average pooling, multiple features under each magnification are merged into a single feature. Then the tabular features are concatenated with the features of different magnification images, and the fully connected network layer is used to perform feature fusion to obtain cross-modal fusion features.

#### Recalibration of MIL representation based on cross-modal fusion features

In the network branch of image features, the features under each magnification need to be processed separately to filter out effective features. Here, the cross-modal fusion features are concatenated with the patch features at each magnification, and the fully connected network layer is used for local and global feature fusion. The merged features are processed using a multi-layer fully connected network followed by the activation function to obtain the weight that represents the relative importance of each patch. For the feature of each magnification, the weight is multiplied back to the feature of the patch, and then all the patch features are summed to obtain the embedded feature representation of the pathological image under the magnification.

#### Recalibration of tabular learning-based representation of the clinicopathological parameters based on cross-modal fusion features

In the network branch of the tabular feature, after the cross-modal fusion feature passes through the fully connected network layer, the activation function is used to obtain the recalibrate factor of the branch feature, and the tabular feature is recalibrated.

#### Fusion of the recalibrated features for final prediction

After the previous processing, the fused table embedding feature and the embedding feature of the image under each magnification have been obtained. In the classification output part, the recalibrated tabular features and the recalibrated image features are concatenated together, and the multi-layer fully connected network is used as the classifier of the entire model to predict the lymph node metastasis of breast cancer and obtain the probability of metastasis.

#### Model training and validation

Because WSIs in the MIL method have a variable patch number (i.e., from less than 10 to more than 800 patches in individual WSI), the model was designed to accept different instance numbers as input. During the model training, Adam algorithm with momentum 0.9 and weight decay 5×e^-4^ was employed. The learning rate was set as 0.02. Data augmentation includes rotating, flipping, changing brightness and contrast. The best model was selected on the validation set of the single-center cohort collected by the Fourth Hospital of Hebei Medical University. The model performance was evaluated on the test set of the single-center cohort collected by the Fourth Hospital of Hebei Medical University. We further evaluated the model generalizability on the external dataset collected by four multiple centers in Hebei Province. Label smoothing was used to prevent the model from learning the label-related bias. A weighted sampling method was applied to the distributed training to achieve a balanced distribution of samples across the four categories in the training dataset. The final loss was computed as follows:

1 $${\mathcal{L}}{\mathscr{=}}{\mathscr{-}}\mathop{\sum }\limits_{{\rm{i}}=1}^{{\rm{n}}}\left\{\left(1-{{\varepsilon }}\right)\left[-\mathop{\sum }\limits_{{\rm{y}}=1}^{{\rm{K}}}{\rm{p}}\left({\rm{y}}|{{\rm{x}}}_{{\rm{i}}}\right){{\log }}{{\rm{q}}}_{{{\theta }}}\left({\rm{y}}|{{\rm{x}}}_{{\rm{i}}}\right)\right]+{\rm{\varepsilon }}\left[-\mathop{\sum }\limits_{{\rm{y}}=1}^{{\rm{K}}}{\rm{u}}\left({\rm{y}}|{{\rm{x}}}_{{\rm{i}}}\right){{\log }}{{\rm{q}}}_{{{\theta }}}\left({\rm{y}}|{{\rm{x}}}_{{\rm{i}}}\right)\right]\right\}$$

where ${{\rm{q}}}_{{\rm{\theta }}}({\rm{y|}}{{\rm{x}}}_{{\rm{i}}})\,$ denotes the predicted likelihood from the model for sample ${{\rm{x}}}_{{\rm{i}}}$, ${\rm{n}}$ is the number of samples, ${\rm{K}}$ is the number of candidate labels, and ${\rm{\varepsilon }}\in [0,1]$ is a weight factor. In practice, ${\rm{u}}\left({\rm{y}}|{{\rm{x}}}_{{\rm{i}}}\right)$ is not dependent on data; thus, we set ${\rm{u}}\left({\rm{y}}|{\rm{x}}\right)=\,\frac{1}{{\rm{K}}}$.

The training process was conducted on a standard workstation with eight NVIDIA TESLA P40 GPUs. We applied the Adam optimizer (momentum 0.9, weight decay 5 × e^−4^, and batch size 8) to minimize of the loss. The learning rate of tabular model part, the classifier and the rest of the model parameters were set to 1 × 10^−2^, 1 × 10^−3^, and 1 × 10^−4^, respectively. The model was chosen based on the performance of the validation set. At validation or test stage, the paired data (patches from the WSI and clinicopathological parameters) were fed into the model once, and the learnt multi-modal multi-instance fusion module integrated them with optimized attention mechanism to generate the final representation for each patient.

#### Model interpretation

Both MIL and tabular methods are based on the attention mechanism. We investigated the feature importance based on the learned weights of the instances in the MIL and the features of the clinicopathological parameters after the joint learning process.

### Statistical analysis

The area under the receiver operating characteristic (ROC) curve was calculated using the pROC in R (version 3.6.1), and the Delong test was applied to compare ROC curves. Cutpointr was used to estimate the optimal cutoff points of the ROC curves. The Wilcoxon rank-sum test was used to compare the signatures. Pearson correlation coefficients were used for the correlation analysis. *P* values < 0.05 were considered statistically significant, and all *P* values were two sided.

### Reporting summary

Further information on research design is available in the Nature Research Reporting Summary linked to this article.

## Supplementary information

Reporting Summary

## Data Availability

The data can be used only for "non-commercial" purposes and under the permission of the corresponding author.

## Code availability

The code can be used only for “non-commercial” purpose and under the permission of the correspond author. The code for analysis and experiments were publicly available here https://github.com/TencentAILabHealthcare/BreastCancerLymphNodeMetastasis.
